# The habilitation nursing of children with developmental disabilities—Beyond traditional nursing practices and principles?

**DOI:** 10.3402/qhw.v9.23106

**Published:** 2014-03-21

**Authors:** Johanna Olli, Tanja Vehkakoski, Sanna Salanterä

**Affiliations:** 1 Department of Nursing Science, University of Turku, Turku, Finland; 2 Department of Educational Sciences/Special Education, University of Jyväskylä, Jyväskylä, Finland

**Keywords:** Disability, children's neurological ward, nurse, principle, case study, content analysis

## Abstract

Research-based descriptions of the contents of the habilitation nursing of children with developmental disabilities are lacking. The objective of this qualitative study was to describe the habilitation nursing of children with developmental disabilities in a Finnish children's neurological ward. In addition, the purpose was to outline the principles that directed the nursing functions (which consisted of various nursing interventions). The data collection included observation, a retrospective think-aloud method with video-taped nursing situations, the nursing records, and an open-ended questionnaire. The data were analysed with a qualitative content analysis of the manifest and latent content. The findings show that habilitation nursing in a children's neurological ward consists of assessing the child's skills, supporting the child's development, and collaborating with the child's immediate adults. When implementing those functions with nursing interventions, the nurses demonstrated four principles: client-originated and professional-originated principles, and individual-centred and community-centred principles. Becoming conscious of these principles and the theoretical frameworks behind them enables the development of a nursing science–based model for habilitation nursing.

The habilitation^1^ nursing of children with developmental disabilities is a rarely researched area (Griffits, Bennett, & Smith, 2007). The habilitation of children with developmental disabilities should be begun as early as possible in order for them to put their evolving capacities to good use, to enjoy their rights and liberties on an equal basis with other children (United Nations, 2006), and to ensure full participation and equality (World Health Assembly, 2005). In many countries, registered nurses are part of the multiprofessional team which takes care of the habilitation of children with developmental disabilities. Their part in children's habilitation should be studied in order to develop habilitation nursing with principles based on nursing science.

Even though no research exists on the practices of the habilitation nursing of children with developmental disabilities, it is reasonable to look for similarities in rehabilitation nursing practices for adult patients. Combining nursing and rehabilitation seems to be problematic for nurses (Long, Kneafsey, Ryan, & Berry, 2002; Pryor, Walker, O'Connel, & Worral-Carter, 2009). One reason for this is the practice of working in a multiprofessional team (Pryor et al., 2009), which might introduce nurses’ ambiguity in the nurse's role when carrying out their part of the whole team's rehabilitation task. In addition, rehabilitation nursing differs from traditional nursing, which might emphasize taking care of patients’ hygiene and medication more than supporting their independence (Long et al., 2002; Pryor et al., 2009).

The difficulty of combining nursing and rehabilitation may be related to the different theoretical frameworks behind rehabilitation and nursing. According to rehabilitation nursing studies, the traditional nursing framework includes the thought of “caring for” and “doing for” the patient (Long et al., 2002), where the patient's active participation is not considered important and the nursing process is not long-term goal oriented (Pryor et al., 2009). However, over the past several decades nursing science literature has described a framework in which the aim of nursing care is to help the patient towards independence and a life suitable for that patient (Eriksson, 1994, 2001; Henderson, 1964; King, 1981; Watson, 1979). According to these theorists, the basis for this kind of thinking is a multidimensional conception of human beings and health in which important issues are the patient's responsibility for his/her life and the patient's right to decisions concerning his/her own life. This aim is reached through a care relationship (in which the patient is respected and listened to) as well as a caring environment and sense of belonging to a community (Eriksson, 1994, 2001; Henderson, 1964; King, 1981; Watson, 1979). An essential idea of this way of thinking is that in a good care relationship a human being finds his/her own resources (Eriksson, 2001).

The framework of rehabilitation has traditionally been based on the medical (individual) model of disability, in which disability is seen as a problem in an individual's features, and rehabilitation as modifying the individual by the cultural norms of normality (Oliver, 1996). A different perspective is offered according to the social model of disability (Abberley, 1987; Oliver, 1996; Scotch & Schriner, 1997), in which disability is considered a condition caused by cultural prejudices, inadequate societal services, and physical environments which are not able to meet the different physical and mental human variations (e.g., impairments) which appear in any particular community. This leads to seeing the whole of society (and changing the circumstances) as the object of actions to be taken with the aim of full integration/inclusion into society (Oliver, 1996; World Health Organization, 2007).

In addition to the frameworks of rehabilitation and nursing, the general frameworks of child care and education are present in habilitation nursing. In health care, attitudes towards children have long been based on a framework which emphasizes children's vulnerability and need for protection. When acting according to that framework in nursing practice, adults decide on children's issues often without listening to the children (Coyne & Callagher, 2011; Kilkelly & Donnelly, 2006; Runeson, Hallström, Elander, & Hermerén, 2002). It seems that family centredness, which has for long been an essential and acknowledged principle in paediatric nursing, might have quite often meant parent-centredness in nursing practice and research (Mikkelsen & Frederiksen, 2011). This is problematic because even parents cannot always listen to their own child or to be on his/her side (Runeson et al., 2002). Not listening to children, at least to small children, has been the general practice in nursing research as well, where informants on the child's issues have usually been parents, nurses, or older children.

During the past decade some signs of a framework emphasizing children's agency have appeared in nursing research, in which even small children have been considered to be adequate informants – for example, in the studies of Kortesluoma & Nikkonen (2004) and Salmela, Salanterä & Aronen (2009). This development is parallel to the childhood studies’ view of children and childhood. According to that view, even small children are active social agents who want and are able to influence their own life's decisions (James & James, 2004; Mayall, 2002). Children also have the right to be heard and to be taken seriously in these decisions—according to the Convention on the Rights of the Child (United Nations, 1989) and many countries’ laws (the Constitution of Finland, the Social Services Act/Denmark, the Child Welfare Law/Norway, the Child Protection Act/Iceland, the Social Services Act/Sweden, and the Children Act/UK).

Different theoretical frameworks appear in various conscious and unconscious principles guiding the implementation of nursing care. Neither these principles in children's habilitation nursing nor the nursing functions in children's habilitation have been studied. The aim of this study is to fill this gap by describing the nursing functions in a Finnish children's neurological ward and by examining what principles appear in the nursing interventions in which the functions are implemented. Producing a detailed and theoretically argued description—of which exists only tacit knowledge—may promote understanding of the practices, add awareness of the meaningful principles, and act as a ground for developing practices.

## Methods

### Design

Our study design was a qualitative case study which is suitable for a topic such as habilitation nursing that has been little researched or not researched at all (Yin, 2003) and because, with a case study research method, detailed information can be gathered about both the informants’ (nurses) thinking and their actions (Polit & Hungler, 1995). In collecting the data, mixed methods were used in order to obtain both the participants’ and the researcher's perspectives. The included observation (OBS), interviews by using a retrospective think-aloud method while watching videotaped situations (VID), nursing records (REC), and an open-ended questionnaire (QUEST) (Table I).

**Table I:** Data collection methods.

Observation	- Semi-structured
(OBS)	- Passive
	- Participatory
	- Five workdays (40 h)
Interview and video (VID)	- Think-aloud method while watching videotaped sessions:
	Playing (4)
	Eating (2)
	Bathroom (1)
	Testing (2)
Reading the	- 23 pages daily records
nursing records	- 1 page nursing plan
(REC)	- 1 page nursing summary
Open-ended	- By e-mail to 5 nurses
questionnaire	- 3 open-ended questions:
(QUEST)	1. What is the habilitation nursing of children with developmental disabilities?
	2. What are the aims of the habilitation nursing of children with developmental disabilities?
	3. What interventions are involved in the habilitation nursing of children with developmental disabilities?

### Setting

The case of this study was habilitation nursing, and the context of the case was a children's neurological ward in one Finnish public special healthcare hospital. Extreme case sampling (Patton, 2002) was used when selecting a ward, which was exceptional in that the nurses there used many different assessment and habilitation methods. The ward that was selected also had an exceptionally good staffing level: each nurse took care of one family at a time.

The ward operated only during the weekdays and only during the day. Its patients were children aged 0–18 years with different kinds of neurological illnesses or impairments. Typically, a child with developmental disability visited the ward for 5 days once or twice a year. During these hospital periods the child's development was assessed, and his/her habilitation was designed by a multiprofessional team. The team consisted of a neurologist, a neuropsychologist, a physiotherapist, a speech therapist, an occupational therapist, a social worker, and a registered nurse. Primary nursing care was the prevailing practice in this ward, which means that every child had his/her own nurse.

### Data collection

The observation was carried out over five of the key informant nurse's workdays. Intensity sampling (Patton, 2002) was used when selecting the key informant nurse on the basis of the preliminary observation on the chosen ward. The key informant nurse was a paediatric nurse whose work intensely manifested the phenomenon of habilitation nursing by providing an information-rich (but not exceptional or highly unusual) case. She had worked 10 years on the ward and had a wide range of experience in nursing care.

The observation period included the entire hospital stay of one child. Typical case sampling was used when selecting the child, a typical patient of the chosen ward: a preschool-aged child with the most common diagnosis in the ward: F83, that is, “mixed specific developmental disorders” (World Health Organization, 1992). The child's hospital period was planned to include diverse interventions of habilitation and health care, as usual on the chosen ward. We selected the first child who met the aforementioned criteria and was coming to the ward during the planned observation period. The observer (the first author) followed the nurse in all her actions (excluding her private pauses and visits outside the ward) and documented all her actions using semi-structured forms (about what happened and where and who was present). The forms were designed for this study and revised after 1 day of preliminary observation.

In addition to direct observation, the key informant nurse was also videotaped with the child in different situations (Table I), which were selected with the nurse according to how typical they were for nursing on this ward. These videotapes were shown to the nurse after each workday and the nurse was interviewed about her actions and thoughts in those videotaped situations. The interview was carried out using a retrospective think-aloud method (Van den Haak, De Jong, & Schellens, 2003) while watching the videos.

Data relating to the nurse's thoughts on the subject was also collected by reading her nursing records on the child. Furthermore, an open-ended questionnaire was sent to all the nurses (n=5) in the ward via email except for the head nurse, who had only worked at the ward for a short time. All the nurses were specialized in paediatric nursing and had more than 5 years of work experience in the children's neurological ward.

### Data analysis

In the first analysis, we formed a case description to answer a question about the functions of the habilitation nursing of children with developmental disabilities. In forming the case description we processed all data sources together, as Yin (2003) suggests. We analysed the manifest content of the data with an inductive content analysis (Graneheim & Lundman, 2004). As a meaning unit, we used an action or utterance (e.g., “The nurse asks and signs: ‘milk or water?’”). Then we categorized the meaning units into wider unities: categories (e.g., asking the child questions), sub-themes (e.g., supporting communication), and themes (e.g., supporting the child's development). The level of abstraction rose during the categorization.

We later returned to the data and wanted to find out what kind of thinking (called as professional principles) directed the implementation of the nursing functions. We then analysed the latent content of the whole data (Graneheim & Lundman, 2004) according to the example below (Table II). The themes that we called principles emerged inductively from the data, but represented more theoretical interpretations that were based on our understanding of the existing practices and abstraction of the data. In the analysis, the parts and the whole were reflected against each other, as Graneheim and Lundman (2004) suggest. This means that each meaning unit was examined from the perspective of the whole, so that the interpretation of a single utterance would be relevant to the data of a particular participant as a whole. The same meaning units were used in several sections if we found many possible underlying meanings. An example of this kind of utterance and also of the process of content analysis is illustrated in Table II. In addition, we analysed the linguistic features of the nurses’ utterances in order to obtain deeper understanding of the meanings.

**Table II:** An example of the analysis of latent content (open-ended questionnaire data).

Meaning unit	Condensed meaning unit	Interpretation of the underlying meaning	Sub-theme	Theme
The idea of nursing is to find and to make observations about the child's development by using different methods as aids. Observation is the most important [method] where the child's skills are compared to the developmental skills of a so-called “normal” child. (QUEST*)	The most important aspects of the child's assessment are being observed by professionals and being compared to “normal” children.	An assessment based on a professional's knowledge gives the most important information about the child.	The basis of the assessment is the professionals’ perspective.	Professional-originated principle.
The idea of nursing is to find and to make observations about the child's development by using different methods as aids. Observation is the most important [method] where the child's skills are compared to the developmental skills of a so-called “normal” child. (QUEST)	The most important aspects of the child's assessment are being observed by professionals and being compared to “normal” children.	What is important in the assessment important is assessing the child's skills (not the influence of the environment or other people).	The target of the assessment is the child only.	Professional-originated principle.

* For the abbreviations of the data sources, see Table I.

### Ethical considerations of the study

Ethical issues were considered carefully throughout the study, especially concerning the possible advantages and disadvantages for the child involved. The study was designed so that the normal care could be carried out and no extra harm to the child was caused. In addition, the study is considered to be valuable in developing habilitation nursing.

Each adult participant received written information about the study. Written consent was obtained from all of them after discussing the voluntary nature of the project and the right of any of the participants to discontinue the study if they so desired, the purpose and methods of the study, and its possible disadvantages. The parents of the child gave their consent on the child's behalf, because of the child's young age. The parents presumed that the presence of the observer and the camera would not disturb the child in nursing situations. Moreover, the observer did not see any reaction from the child which would indicate the child was disturbed about the data collection. This can be interpreted as the child's assent for the data collection—even if a better way would have been to directly ask his/her informed assent via augmentative and alternative communication methods, for example, by using picture cards.

The anonymity of the participants was guaranteed by not following the nurse outside of the children's neurological ward and by writing the research report so that readers cannot identify the participants. Careful consideration was given for protecting the privacy and human dignity of the participants in each phase of the study. The study was approved by the hospital authorities.

## Findings

The nursing in the children's neurological ward included several distinct functions: assessing the skills of the child, supporting the child's development, and co-operating with the child's immediate adults. In addition, part of the nurse's work was to take care of the child's basic needs and physical health (such as administering medication) and practical arrangements (such as preparing the room for a team meeting). The latter three functions are not handled in this article because they are more generally associated with nursing, instead of specifically with habilitation nursing.

Assessing, supporting, and co-operating have three common features in the children's neurological ward. First, the nurse works closely with the child's and the family's everyday life. The nurse takes care of the children's group during mealtime and playtime, which resembles home and day care activities more than standardized testing situations with other professionals (e.g., a speech therapist or neuropsychologist) in their offices. Therefore, the nurses are able to observe and assess the child in circumstances that most clearly parallel the child's normal living. They also have opportunities to plan and try habilitation ideas with the child and parents in circumstances that are closely related to those of the child's everyday life.

Second, nurses act as intermediaries between the family and the multiprofessional team when assessing the child, supporting the child's development, and co-operating with the child's immediate adults. Nurses meet at least one parent every day when the parent brings the child to the ward or picks him up (if the parent does not stay on the ward with the child the entire time). In addition, the nurses disseminate vital information about the child's actions to team members.

Third, habilitation nursing in the children's neurological ward appears to be fairly autonomous even though it includes close co-operation with a multiprofessional team. The nurses plan and implement nursing interventions mainly independently.

The functions of habilitation nursing are implemented according to several different principles. These principles describe both whose perspective is the basis of the habilitation (client- or professional-originated principles) and to whom the habilitation is targeted (community- or individual-centred principles). The professional-originated principle refers to preferring the knowledge of professionals (e.g., standardized test results; normative criteria for age-appropriate skills) to the knowledge produced by the child or the parents emphasized in client-originated principle. By an individual-centred principle we refer here to considering the skills of the child as a primary or even as the only target of the habilitation. Conversely, the community-centred thinking means seeing other people and the environment as a source of children's problems and therefore, as a possible solution of them and similarly, as a target for habilitation as well. All four of these principles were seen in every nurse's actions or perceptions; they drew on them to a varying extent in carrying out the functions of the habilitation nursing. Next, we will describe the implementation of habilitation nursing's three functions and the principles behind their implementation.

### Assessing the skills of the child

One of the functions of habilitation nursing in a children's neurological ward is to assess the skills of the child, partly independently and partly together with the multiprofessional team. In the independent assessment the nurse uses several different methods as nursing interventions: structured assessment methods (the checklist in the Portage Guide to Early Education, the Childhood Autism Rating Scale, the Psychoeducational Profile-Revised, ICD-10), an unstructured assessment of everyday situations in the ward (e.g., eating, play), and the collection of information from the child's family and the day care personnel. Co-operation with the multiprofessional team occurs both in informal discussions during the child's hospital period and in the team meeting at the end of the period.

The constant assessment in every situation is also visible in the nursing records, as the following data extract from the nurse's notes on a free playing situation shows: “Free play with animals did not develop into narrative play, instead the child gets stuck in twiddling the dinosaur's tail and the knot on the horse's tail.” (REC) The example above demonstrates about professional-originated and individual-centred principles. Even in free play situations, the nurse's primary function is to assess the child, not to take up a position in a dialogical intercourse with the language of play. Appraisal according to normative criteria and expectations about age-appropriate development (shared by professionals) as a central issue is seen in the example. Narrative play is described from the professional perspective as the correct form of play, which is not actualized. This perspective is expressed through negation “did not develop into narrative play,” and the actualized play was expressed with negatively toned verbs “get stuck” and “twiddling.”

When the nurse uses assessment methods based on normative criteria, the child has no possibility to influence what is assessed about him/her. However, the client-originated principle appears when the child is allowed to choose his activities in the playroom or influence the amount or content of activities in other situations. For example, in the following extract, the nurse takes the child's interest seriously and is flexible in a formal assessment situation, which seems meaningful from the child's perspective: “Well, in this test it would have been enough to cut one piece of paper with scissors, but I let him go on with cutting, because he obviously seemed to like doing that” (THAL).

Occasionally, hints of community-centred principles are observable when the nurse observes the effects of her actions on the child and adapts her actions accordingly. The nurse's actions vary from purposeful passiveness to active guidance of the child.

The reason that I didn't open the door right away, it was because I wanted to wait and see if the child would ask for help. (THAL)

I painted that face because I wanted to see if he would be excited by it, because I didn't get him to draw any kind of human figures in the test [PEP-R], so I thought that this technique might please him more and he would get excited about it. (THAL)

### Supporting the child's development

The second part of the functions of habilitation nursing is support of the child's development. The nurse supports the child's communication, independent initiative in activities of daily living (ADL), executive functions, and motor skills. Table III shows that the nurse carries out a large repertoire of various interventions, from verbal hints to hands-on guidance and from praising to setting strict rules. Augmentative and alternative communication is widely used. Additionally, the nurse supports the child's development by searching and testing suitable habilitation means and assessing the suitability of assistive devices.

**Table III:** The nursing interventions for supporting the child's development in this study.

Communication	Independent initiative in activities of daily living	Executive functions	Motor skills
**Giving positive feedback**	**Giving positive feedback**	**Guiding the child to perceive time and space with pictures**	**Guiding the child to grasp objects properly**
The child names milk and margarine; the nurse praises him. (OBS)	The child brings the plate to the kitchen; the nurse praises him. (OBS)	“So, the child has a pictured schedule, and the aim is to teach the child about things happening in a certain order, before and after.” (VID)	The nurse prompts the child to hold the fork properly. (OBS)
**Asking the child questions**	**Encouraging independent action**	**Structuring actions with pictures**	**Using physical guidance**
The nurse asks the child to look at the next picture, and asks if he knows what it is. (OBS)	The child pushes the plate to the nurse. The nurse says: “No, you will bring it!” (OBS)	The nurse shows the child how to wash his hands with a picture series. (OBS)	“And I guided him by the hand, because he turns the fork in a strange position.” (VID)
**Speaking clearly**	**Giving time for the child's own action**	**Making clear rules and predicting situations**	**Trying and assessing assistive devices**
“The task is given as slowly and as simply as possible.” (VID)	The nurse waits to see if the child will take the napkin to the rubbish bin. (OBS)	“And here is anticipation for what comes next, so that will prepare for this action to end soon; the change is expected.” (VID)	“Lately the issue with assistive devices has taken on a larger role, so we have to put different kinds of gadgets to the test and assess if they are working or not.” (QUEST)
**Using pictures**	**Guiding the child verbally**	**Guiding the child to make his own choices**	
The nurse shows pictures of milk, juice, and water to the child and asks which one he wants to drink. (OBS)	The nurse prompts the child to put his shoes and mittens in the closet. (OBS)	The nurse lets the child choose between two different ice creams. (OBS)
**Using gestures**	**Guiding the child physically**	**Supporting his attention with pictures**	
“Well, there I have wanted to strengthen my speaking also with a gesture.” (VID)	The nurse guides the child from his shoulder, shows the knob of the toilet bowl, and guides the child's hand; he pushes the knob. (OBS)	The nurse guides the child by the hand to the schedule and explains what they have done, guides him to turn the picture over, and guides him back [to the schedule] when he is leaving, and asks him what the next picture is. (OBS)
**Using signs**	**Practicing skills together**		
The nurse says and signs that the child can now play. (OBS)	The nurse and the child butter the bread together. (OBS)
**Using objects**			
The nurse asks the child to point [at a water pot or milk carton] when he can't say [if he wants milk or water]. (OBS)

What is noteworthy is that some kind of support is included in every action taken by the nurse, along with other purposes. Instead of separate habilitation situations, the habilitation is thus included in the ward's everyday actions, for example, eating situations:

At the dining table the child is looking at the drink pictures and then looks at his mug. The nurse won't give the drink to him. Yesterday they practiced how to show pictures when making a choice, and it worked.

The nurse: Well, he looks at the drink pictures. I assume that he would have wanted to have a drink, but when I didn't offer that to him, he got nervous and started to flapping [his hands]. He looked repeatedly at the pictures and his mug. Researcher: And why did you end up not giving him a drink, even when he repeatedly looked at the pictures, but wasn't able to do anything but flap?

N: I was after the thing that he has to ask, he has to give some sign of what he really wants. By not interpreting his glances, we try to get him somehow enlightened to the idea of the power involved in communication and self-expression, that you really get something for yourself by using such power.

[The videotape goes on. When the nurse does not say anything about the next episode, the researcher asks about it.]

R: So, you guide then his fork …?

N: I guide the fork. He would eat only that mash, which is also a good thing, but yes, I guide him by the hand, so that he will taste everything that is on the plate.

N: And I guided him by the hand because he turns the fork in a strange position. So I tried holding his fork in a position where the food really stays there. — I let him practice pouring [milk] into a mug, so we try to strengthen independence skills in every action. (THAL)

The extract shows that the nurse balances between reinforcing the child's independent initiative in the ADL and guiding the child to eat a wider variety of food or to use the cutlery in the right way. When reinforcing the child's ADL skills, the nurse gives the child space to do things by himself (“I let him practice pouring [milk] into a mug”), whereas in promoting healthier eating the nurse turns to physical guidance (“but yes, I guide him by the hand, so that he will taste everything that is on the plate”).

In addition, when the nurse gives food to the child, she not only takes care of his basic needs by giving nutrition but also supports his communication skills by asking him to use a picture card to say what he wants. Aiming at the professional-originated developmental target goes so far that the nurse pretends not to understand his subtle hints (e.g., she does not give him a drink “by interpreting his glances”). Instead, the nurse waits for precise self-expression (“he has to give some sign of what he really wants”).

A client-originated principle emphasizing the child's perspective is rarely seen in supporting the child's development. For example, in the previous data extract the nurse strives to emphasize the importance of self-expression (so that the child will learn to express himself also with strangers), yet the aims and ways of communicating are chosen by adults.

In addition to professional-centredness, supporting the child's development is mainly based on an individual-centred principle, in which revising the child's skills is primary. However, occasionally community-centred principle is apparent in the nurse's actions, when the influence of others’ actions on the child's performance is acknowledged:

Modelling and gestures that support communication are helping. (REC)

He managed to put his clothes on, when he got one piece of clothing on at a time. (REC)

In both examples, the contextuality of the child's skills is acknowledged: skills do not always emerge in the same way, but vary in relation to the environment and the choices of others (e.g., the nurse's choice to use modelling and gestures). In order to be able to guide the child in an optimal way, the nurse observes her own actions’ influence on the child and changes her actions accordingly (e.g., gives clothes to the child one piece of clothing at a time). In this kind of thinking, which emphasizes interventions towards the community surrounding the child, the deficiencies of the child's actions are not reduced to the child's attributes, but also to the adults’ abilities to guide the child according to his needs.

### Co-operating with the child's immediate adults

The third part of the functions of habilitation nursing is the support and education given to the child's immediate adults (such as parents and day care personnel) through several different nursing interventions. Immediate adults are those individuals who might have to change their actions to help the child join in. The support is focused on parents in this study. Forms of support include taking care of the parents’ well-being during the hospital period (e.g., providing opportunities to eat and rest) and creating an empowering relationship by listening, encouraging, and using individual means of support.

Education is focused both on parents and day care personnel. Educating the day care personnel occurs in telephone discussions and in meetings after the child's hospital period. Educating the parents is implemented in small daily doses, both informally in everyday situations in the ward and more formally in the separately conducted discussions.

And then it [habilitation nursing] is informing about social benefits as well, and camps, courses, and that kind of thing. And then it is of course that kind of modelling, it is doing together, it is like demonstrating, so that I show the family how I work with your child in this and this situation. It is about offering alternatives, if this system is not working, could we try it this way. And throughout that it is then making choices. (QUEST)

The extract shows the range of interventions included in the nurses’ co-operation with the child's immediate adults: teaching by giving information, making suggestions, and modelling specific behaviours. In addition, co-operation is “doing together”; by listening to the parents’ needs, the nurses try to figure out the best habilitation means for the child together with the parents.

In their relationship with the parents, the nurses implement the client-originated principle more than they do with the children. All but one of the nurses mentioned listening to the parents in their e-mail questionnaire answers, whereas only one mentioned listening to the children. For example, the nurses consider the parents’ perception of their child and his/her need of support as important, as is seen one nurse's answer:

[Nursing is] discussing with parents about the child's situation – the background knowledge and parents’ perception about his/her development, growth, and strengths. (QUEST)

The parent-originated principle is visible especially in those informal education situations in which the nurse has not strictly planned the outline of for the discussion beforehand; instead, the discussion is shaped flexibly during the interaction. In these cases the nurse seizes on subjects arising from the current situation and develops ideas for supporting the child's development with the parents.

Subjects for informal education arise from three kinds of situations. The first are those, in which the nurse, the parent, and the child are in the middle of the ADL, for example, the child getting dressed. The nurse has an idea while watching the child's actions and asks the parent how dressing situations are managed at home.

The mother and the nurse prompt the child to get dressed. He wanders around the room.

Nurse: Does he manage to get himself dressed at home in the mornings?

Mother: No, we have to dress him, otherwise nothing's gonna work.

Nurse: Well, there is that goal, to get him to understand, that time is limited.

The nurse mentions using an egg timer [which might help the child to understand the limited time] and putting the child's clothes in the right order. (OBS)

The second type of informal education discussions occur during small talk and are not related to the current situation. For example, the mother and nurse are engaged in small talk while the child is eating, and the mother happens to mention something about television. The nurse then asks about the child's amount of daily television watching and gives advice about it.

Third kinds of informal education discussions occur during planned discussion situations, if the nurse allows the discussion to deviate from her original plan, such as is in this summarizing discussion on the day of departure:

Nurse: Strengthening the activities of daily living is just that habilitation [that the child needs], and so is demanding contact. We should quit guessing and wait for the child's needs to be heard.

Mother: If he only could say [what they are]

Nurse: I can give you to take home a toilet picture, which could be on view at home. The problem is that he doesn't have a way to express; would that picture provoke him to remember? In the beginning you show the picture when going to the toilet, then he might notice it by himself.

Father: I wonder if he would go to the toilet in the morning if I didn't take him there?

Nurse: Try, just give him instructions from a distance, if necessary.

Father: Yeah, it has become a routine …

Nurse: Yeah, it's good to remind oneself of that. (OBS)

In the example the nurse guides the parents by giving a concrete tip about strengthening the child's communication skills by using a picture of a toilet. The nurse's advising turn takes the form of a suggestion (“I can give you”). She is also directing the parents to assess the functionality of the idea with her question “would that picture provoke him to remember?” The father joins along to the discussion with the nurse, and it becomes a dialogue, in which they together create ideas for habilitation.

A professional-originated principle is seen especially in more formal, scheduled situations with parents in which the nurse reviews the results of the structured assessment methods (e.g., the results from the Portage checklist), in which cases the interaction remains almost monological. Further, in their answers to the e-mail questionnaire the nurses often describe educating parents as the unilateral provision of information from a professional, as in this data extract: “[The aims of habilitation nursing are] to find the means of habilitation and to get the parents to believe in them and implement them” (QUEST).

## Discussion

In this study, the functions of habilitation nursing of children with developmental disabilities were described for the first time. In addition, we examined the principles on which these functions were based. These functions of nursing covered a wide range of activity, as traditional basic care and care of the patient's health and diseases were combined with observing and supporting all sectors of the child's development, as well as co-operation with the child's immediate adults.

A strength of the nursing care was the opportunity to get to know the child and the family in everyday interaction situations. The nurses were able to assess the child's activities as well as plan and implement habilitation ideas in circumstances close to the child's everyday life. The nurses were able to create a natural relationship through meeting the children daily. They were also able to obtain a flow of information about the child's life at home and to include parents in the ward's everyday habilitation situations. Previous research has shown the importance of this kind of informal and personal relationship between parents and professionals (Watson, Kieckhefer, & Olshansky, 2006).

Regardless of being so close to the family's everyday life, the functions of habilitation nursing in this data described the dominance of professional-originated and individual-centred principles (Table IV). Both of these principles emphasize the professionals’ perspective, and related interventions targeted to modifying the child are linked to strong normative thinking, in which the child is compared to normative standards, not to the child's individuality and his/her previous development (Spitzer, 2003). Thus may be created a picture of the child as a problem or as an object of interventions, instead of seeing him/her as a subject of his/her life (Vehkakoski, 2003).

**Table IV:** The principles of habilitation nursing.

*Whose perspective is the basis of habilitation?*	
The perspective of habilitation professionals Visible in the implementation of these functions of habilitation nursing: - Assessing the child with methods based on normative criteria - Supporting the child's development and educating the immediate adults on practices determined by professionals → **Professional-originated principle**	The perspective of the parents Visible in the implementation of these functions of habilitation nursing: - Educating the child's immediate adults by listening to them The perspective of the child Visible in the implementation of these functions of habilitation nursing: - Supporting the child's development by emphasizing the importance of self-expression → **Client-originated principle**
*To whom/what is the habilitation targeted?*	
Modifying the skills of the child Visible in the implementation of these functions of habilitation nursing: - Assessing the child by emphasizing the child's skills - Supporting the child's development by modifying the child's skills - Educating the child's immediate adults by concentrating on modifying the child's skills → **Individual-centred principle**	Modifying other people and the environment Visible in the implementation of these functions of habilitation nursing: - Assessing the child and educating the child's immediate adults by noticing the influence of other people's actions - Supporting the child's development by modifying other people's actions → **Community-centred principle**

Seeing the child through his/her impairment is related both to the dominance of the professionals’ perspective and the targeting of habilitation to individuals. According to the earlier research, disabled children do not, however, see themselves primarily through their impairment, nor would they wish others to see them in that way (Asbjørnslett, Helseth, & Engelsrudc, 2013; Connors & Stalker, 2007; Kelly, 2005; Watson et al., 2000). In community-centred thinking, for example, communication problems are regarded as contextual and dependent on both parties’ influence, as Komulainen (2005) suggests. Interventions targeted only to the individual do not have the same kind of effectiveness as interventions targeted also to other people and the environment around the individual. Emerging examples of community-centred interventions were seen in the data, which should be expanded. Examples were visible especially when the nurse consulted the immediate adults, but also in their own actions. Even when the nursing interventions were focused on supporting the child's skills, there were examples of facilitating the child's own agency when the nurse noticed the influence of her own actions and the environment on the child's actions.

The emphases in the principles of habilitation nursing mentioned above (Table IV) refer to the medical model and the theoretical framework which emphasize children's vulnerability and need for protection. They also refer to the “traditional” framework for nursing, in which a good nurse takes care of everything on behalf of the patient. In this study the nurses did not simply do everything on behalf of the patient because the nurses in the children's neurological ward really invest in supporting the child's independence, for example, in the ADL. Instead, the nurses seem to know and decide on behalf of the child (and perhaps sometimes on behalf of the family, too), although both the participation rights of the children and shared care and partnership with parents should be essential and acknowledged principles in paediatric nursing. This study focused more on the child's perspective, since it has been ignored in earlier nursing science literature. The literature has most often focused on the parent's perspective, and the concept of family-centred care has included an assumption that the interest of the child always equals that of the parents (Mikkelsen & Frederiksen, 2011). The infrequency of supporting the agency of disabled children has been noted in previous studies from disability researchers in many countries, for example, in Norway (Bekken, 2014), England (Komulainen, 2012), Northern Ireland (Kelly, 2005) and New Zealand (Higgins, MacArthur, & Kelly, 2009). According to our review of several studies, children's agency is hindered, for example, by considering children as an object of professionals’ actions, by interpreting problems as being caused by the child's impairment, and by communicating on professionals’ terms (Authors, 2012).

The usefulness of the social model on disability has long been emphasized in disability studies, and childhood studies have emphasized the importance of listening to children. If disabled children are to become thriving citizens with full human rights, now would be the time in nursing science and in nursing practice to move towards more client-centred practices with children. Perhaps nursing science—with its long history of collaboration with medicine—could be the innovator that combines medical and social models into a new dialogical model, which notices both the vulnerability and the competence and agency of children (Komulainen, 2012) in theory and in practice. This kind of dialogical model would suit habilitation nursing well because nursing already has the role of being an intermediary between the family and the multiprofessional team, according to this and previous studies (Long et al., 2002). Moreover, the idea of dialogical model is related to those nursing theories, where the central values consist of a care relationship in which the patient is respected and listened to, and where a caring environment and belonging to a community are considered essential (Eriksson, 1994, 2001; Henderson, 1964; King, 1981; Watson, 1979).

The trustworthiness of this study is enhanced by the triangulation of four different data sources. Through purposeful sampling (Patton, 2002) and various data collection methods, a multifaceted picture of habilitation nursing in a children's neurological ward was attained. All of the data sources provided information which supplemented or confirmed the information from the other data sources. However, the trustworthiness of this study is reduced by the fact that the observation notes were in written form and not videotaped. In addition, even richer data would have been collected by interviewing all the nurses, instead of using an e-mail questionnaire. Nevertheless, the very open questionnaire gave the nurses the opportunity to share what they thought was important. The think-aloud method was an especially productive way of deepening the data because it gave very detailed information about the nurse's thought processes (Branch, 2000), which confirmed the observation data. In addition, assessing the preliminary analysis with the key informant enhances the trustworthiness of the study (Yin, 2003).

The transferability of the research findings can be ultimately considered as a decision of the reader and thus, as something that the reader may discover if the research report is evocatively written (Tracy, 2010). The transferability of the findings of this study is, however, justified by our theoretical presumption, according to which nurses’ action and interaction with children is reduced to general nursing culture and practices, and not only to each nurse's individual values. Thus, every piece of interaction may reveal some general patterns and basic structures of social order in the habilitation nursing practices also present within other contexts and other societies as well (Peräkylä, 2004). This is also supported by the identified principles of habilitation nursing (e.g., professional originatedness) which refer to the “traditional” framework for nursing and have also been found in earlier research (Long et al., 2002; Pryor et al., 2009). In addition, the roles which research participants take up (such as nurse and patient) are supposed to be relatively universal category memberships with the same kinds of role expectations in various societies and cultures, although the specific research on nursing positions in the field of habilitation nursing is lacking and further research is thus needed.

The major limitation in this study is that it describes nursing only from the nurses’ perspective, not from the perspective of the child, the family, or the multiprofessional team. In particular, the lack of the child's perspective is similar to the limitations faced by previous research. Moreover, our decision not to ask for the child's consent reflects the values common in habilitation practice and society: the child is not asked. We learned only during the process how important and possible it is to ask for consent also from small disabled children (Cocks, 2006).

In the future it would be significant to clarify the child's point of view as well. For instance, this study addresses questions about why the assessments and plans for habilitation are carried out at hospital (in a strange environment to a child) instead of taking them somewhere nearer to children's and parents’ real life. In addition, it would be important to study interaction practices in habilitation nursing with more detailed methods in order to find which of the nurses’ actions facilitate or hinder the children's participation in their own everyday decisions. In asking the child what he/she thought happened in the nursing situation, alternative and augmentative communication methods could be used with a video recall method.

## Footnotes

1 In this article we use the word “habilitation” instead of “rehabilitation” because with these children it is not about recovering lost skills acquired earlier in life, but learning the skills for the first time.
